# Repurposed clindamycin suppresses pyroptosis in tumor-associated macrophages through Inhibition of caspase-1

**DOI:** 10.1186/s13046-025-03478-5

**Published:** 2025-08-04

**Authors:** Adrian Weich, Johannes Berges, Cindy Flamann, Katrin Bitterer, Krishna Pal Singh, David Chambers, Christopher Lischer, Xin Lai, Olaf Wolkenhauer, Carola Berking, Gerhard Krönke, Shailendra Gupta, Heiko Bruns, Julio Vera, Research Group Macrophages

**Affiliations:** 1 Department of Dermatology, Friedrich-Alexander-Universität (FAU) Erlangen-Nürnberg and Uniklinikum Erlangen, 91054 Erlangen, Germany; 2 Comprehensive Cancer Center Erlangen-European Metropolitan Area of Nuremberg (CCC ER-EMN), 91054 Erlangen, Germany; 3 Deutsches Zentrum Immuntherapie (DZI), 91054 Erlangen, Germany; 4 Department of Internal Medicine 5, Hematology and Oncology, Friedrich- Alexander-Universität (FAU) Erlangen-Nürnberg and Uniklinikum Erlangen, 91054 Erlangen, Germany; 5 Department of Systems Biology and Bioinformatics, Universtität Rostock, Rostock, Germany; 6 Department of Internal Medicine 3, Uniklinikum Erlangen, Friedrich-Alexander-Universität (FAU) Erlangen-Nürnberg, 91054 Rheumatology, Erlangen, Germany; 7 Department of Rheumatology and Clinical Immunology, Charité - Universitätsmedizin Berlin, Berlin, Germany; 8 Biomedicine Unit, Faculty of Medicine and Health Technology, Tampere University, Tampere, Finland; 9 Leibniz-Institute for Food Systems Biology, Technical University of Munich, Freising, Germany; 10 Laboratory of Systems Tumor Immunology, Department Dermatology, Friedrich-Alexander Universität Erlangen- Nürnberg, Hartmannstr. 14, 91052 Erlangen, Germany; 11 Department of Internal Medicine 5, Hematology and Oncology, Friedrich-Alexander Universität (FAU) Erlangen-Nürnberg, 91054 Erlangen, Germany

**Keywords:** Cancer, Caspase-1, Clindamycin, Drug repurposing, Pyroptosis, Tumor-associated macrophages, Systems biology, Network biology, Pharmacophore modeling, Immunotherapy

## Abstract

**Background:**

The metastatic microenvironment is often rich in tumor-associated macrophages (TAMs). In uveal melanoma (UM), high levels of TAMs positively correlate with tumor progression and poorer prognosis. We hypothesize that the immunomodulation of TAMs can remodel the UM tumor microenvironment and make it more susceptible to therapeutic interventions.

**Methods:**

In our work, we designed a novel computational pipeline that combines single-cell transcriptomics data, network analysis, multicriteria decision techniques, and pharmacophore-based docking simulations to select molecular targets and matching repurposable drugs for TAM immunomodulation. The method generates a ranking of drug-target interactions, the most promising of which are channeled towards experimental validation.

**Results:**

To identify potential immunomodulatory targets, we created a network-based representation of the TAM interactome and extracted a regulatory core conditioned on UM expression data. Further, we selected 13 genes from this core (NLRP3, HMOX1, CASP1, GSTP1, NAMPT, HSP90AA1, B2M, ISG15, LTA4H, PTGS2, CXCL2, PLAUR, ZFP36, TANK) for pharmacophore-based virtual screening of FDA-approved compounds, followed by flexible molecular docking. Based on the ranked docking results, we chose the interaction between caspase-1 and clindamycin for experimental validation. Functional studies on macrophages confirmed that clindamycin inhibits caspase-1 activity and thereby inflammasome activation, leading to a decrease in IL-1β, IL-18, and gasdermin D cleavage products as well as a reduction in pyroptotic cell death. This clindamycin-mediated inhibition of caspase-1 was also observable in TAMs derived from the bone marrow of multiple myeloma patients.

**Conclusions:**

Our computational workflow for drug repurposing identified clindamycin as an efficacious inhibitor of caspase-1 that suppresses inflammasome activity and pyroptosis *in vitro* in TAMs.

**Supplementary Information:**

The online version contains supplementary material available at 10.1186/s13046-025-03478-5.

## Graphical Abstract

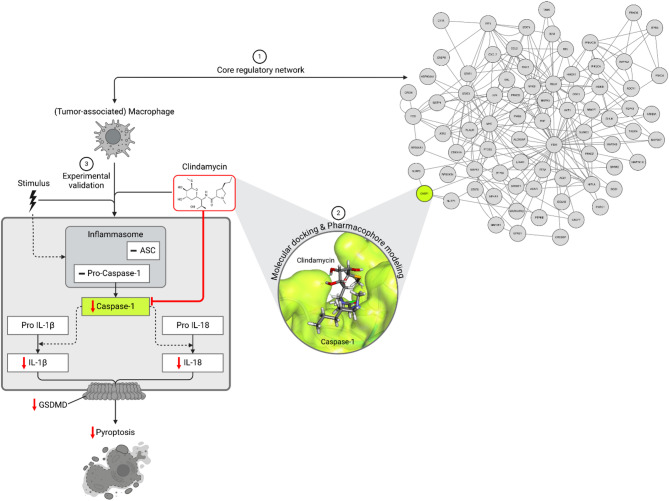

## Introduction

Uveal melanoma (UM), the most common ocular malignancy in adults, has a poor prognosis after reaching the metastatic stage. One factor promoting disease progression in UM are tumor-associated macrophages (TAMs), as TAM enrichment in the tumor microenvironment positively correlates with unfavorable prognosis and shorter survival [29, 62]. We hypothesize that immunomodulation of TAMs can remodel the tumor microenvironment and make tumors more susceptible to therapeutic intervention. Recent findings in pancreatic ductal adenocarcinoma support this by showing that IL-1β, a central effector molecule released by activated macrophages, drives tumor growth, and its inhibition alleviates inflammation [6]. In an effort to identify similar key TAM regulatory factors exerting tumor-critical functions, we developed a combined computational and experimental workflow that selects therapeutic targets for TAM immunomodulation.

The workflow was designed for drug repurposing, i.e., the re-use of approved drugs in new clinical contexts. Drug repurposing leverages prior knowledge about the biodistribution and toxicity thresholds of existing drugs to reduce the time from discovery to regulatory approval [45, 51]. Traditionally, drug repurposing research has been implemented through systematic *in vitro* screenings [31]. Taking advantage of computational biology, one can both accelerate the process and discover new molecular drug interactions. Goody and co-workers, for example, combined a virtual screening of an FDA-approved molecule library through docking and *in vitro* experiments to discover Argatroban’s interference with the interaction between metastasis-associated protein 1 (MTA1) and the transcription factor E2F1 [15].

**Fig. 1:**
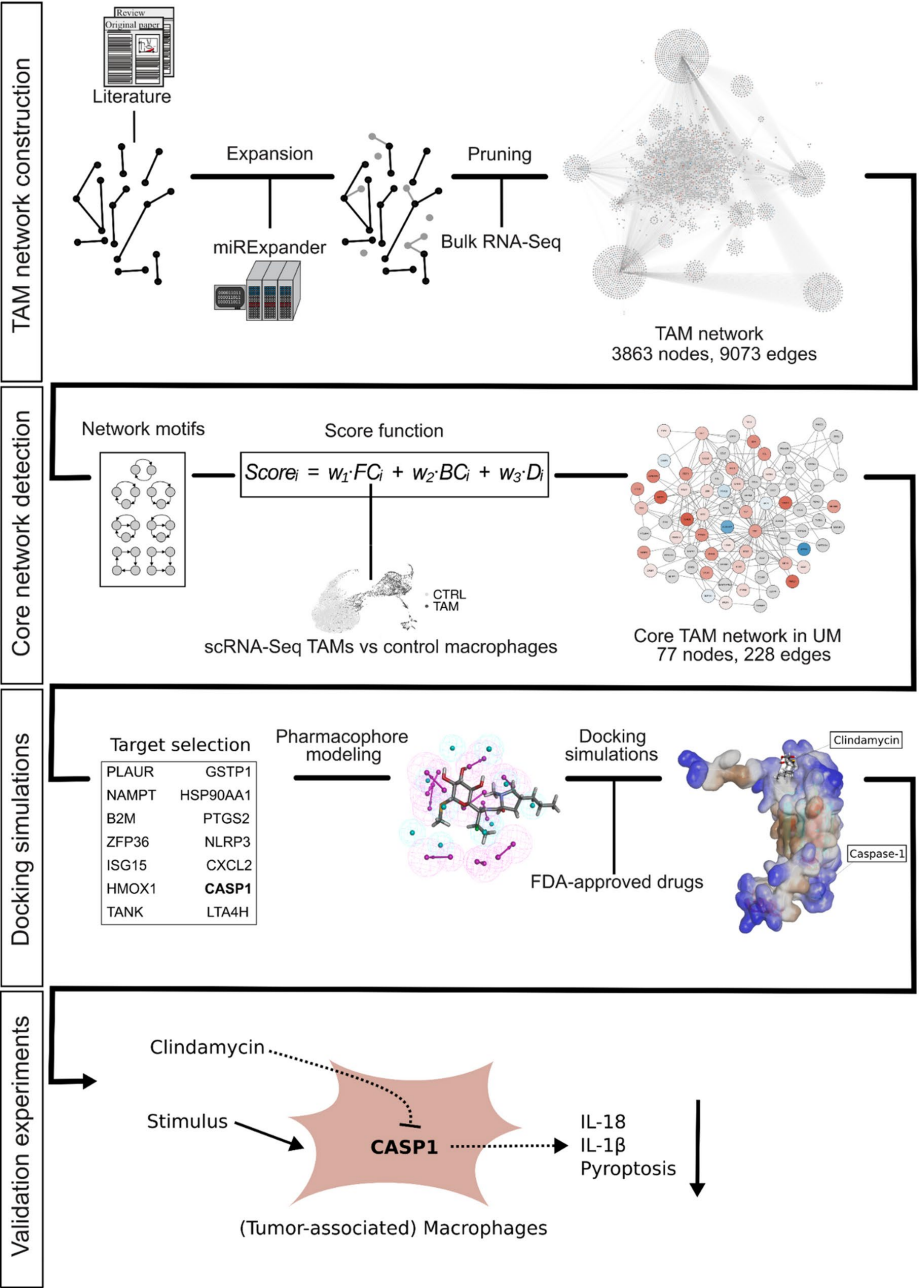
Workflow designed to detect, select, and test molecular targets and drugs for repurposing

Here, we introduce an integrative computational workflow that leverages transcriptomics data, network-based protein target selection, and pharmacophore modeling of an FDA-approved drug library (Fig. 1). We applied the workflow to TAMs in UM as a case study and discovered and experimentally validated a novel and contextually impactful interaction between the antibiotic clindamycin and caspase-1. In our experiments, this interaction inhibits the secretion of pro-inflammatory cytokines such as IL-1β from macrophages, thereby suppressing pyroptosis, a pro-inflammatory form of programmed immune cell death [43]. We also show that the methodology and key results are not limited to UM.

## Materials and methods

### Workflow

To repurpose drugs for the modulation of tumor-associated macrophages (TAMs), we implemented the following workflow (Fig. 1):

1. **TAM network construction**: We collected bulk RNA sequencing data and signaling pathway data from public repositories and literature. The latter was used to construct a regulatory network of possible biological interactions, while the former was used to achieve TAM specificity by projecting expression data onto the respective network nodes.
2. **Core network detection**: We extracted regulatory motifs from the network and ranked them based on their estimated importance for TAMs. For scoring, we aggregated network features (node degree, betweenness centrality) and differential expression derived from single-cell RNA-Seq (scRNA-Seq) data of UM-associated macrophages and steady-state control macrophages.
3. **Docking simulations**: After ranking potential targets from the core network, we generated pharmacophore models of selected proteins, virtually screened them against FDA-approved drugs, and applied flexible docking to assess the drug-target binding.
4. **Validation experiments**: We performed *in vitro* experiments to validate novel drug-target interaction and assess its impact on macrophage behavior.

The individual steps are explained in more detail below.

### Data collection

We obtained publicly available sequencing data from the GEO database. The data used for the network customization consisted of 12 bulk RNA sequencing samples (GSE117970) of macrophages associated to breast or endometrial cancer [7]. The data analyzed for the differential expression consisted of scRNA sequencing results of 8 primary and 3 metastatic uveal melanoma samples (GSE139829) [13] and a collection of samples from healthy joint macrophages (GSE134691) [11].

### Differential expression

Following the analysis workflow of the original publication, we combined all cells from 8 primary and 3 metastatic tumor samples with at least 120 recognized features into one dataset using Seurat (4.4.0) in R (4.05) [20, 60]. To identify and extract TAMs, we used the macrophage identifiers CD68, CD163, and CD14, where at least two markers needed an expression greater than 1 TPM. This stringent criterion was applied to reduce the false-positive rate, thereby avoiding contamination with other cell types. We then combined the TAM subset with the steady-state joint macrophages (CTRL) in one Seurat object and discarded cells in which mitochondrial genes contributed more than 25% of all reads, considering them contamination. After scaling, normalization, and principal component analysis using standard Seurat procedures, we performed batch correction using harmony [28]. Across both groups, TAM and CTRL, we performed differential expression analysis with the “FindMarkers” function, though only including features that were expressed by at least 1% of cells in both conditions. We fed the key characteristics of differential expression for called genes (adjusted p-value smaller than 0.05 after Bonferroni correction) into the core network extraction. To further classify the cells into polarized sub types, we used M1 polarization markers CD14 and IL-1β and M2 polarization markers CD163 and IL-10. The plots were generated using Seurat’s “DimPlot” and “FeaturePlot” functions and ComplexHeatmap [17].

### Network construction

The TAM network was derived from the macrophage network by Wentker et al. [63]. As this network was intentionally focused on the M1-like polarization and TAMs are known to display more plasticity, we supplemented curated M2-like signaling machinery based on literature review [40, 41, 47]. To this end, we manually queried PubMed for terms concerning the M2-like macrophage phenotype, including “M2 macrophage polarization”, “alternative activation of macrophages”, and “anti-inflammatory macrophages”. This information was inserted into the existing macrophage map using CellDesigner (v4.4.2) [14, 27], and each new interaction was annotated utilizing CellDesigner’s MIRIAM capabilities [46] according to the procedure described in Wentker et al. When genes and proteins extracted from literature were of murine origin, we annotated them as their human equivalent after identity translation with the biomaRt R package (2.56.0).

Next, we extended the network automatically with additional molecules and relations taken from the databases miRTARBase (version 6.1) [9], miRecords (version 4.5) [65], HTRIdb (version 1) [4], and TRANSFAC (version 2015.1) [42] using an in-house tool named miRNExpander (https://github.com/marteber/miRNexpander*)* and translated the network into Cytoscape (v3.8.0) [55].

We condensed the expanded macrophage network to a TAM network by pruning it based on RNA-seq data from 12 samples of breast or endometrial cancer associated macrophages (GSE117970). To this end, we combined the RNA-Seq data in R (4.0.5) and transformed the counts to transcripts per million (TPM) using Ensembl transcriptome as transcript length reference (version GRCh37.87). We calculated each gene’s average TPM value across samples and added it to the expanded network. For a node to be preserved, it needed an average TPM above 10 and a non-zero node degree. The obtained network can be browsed and downloaded from www.vcells.net/TAM-drug-repurposing.

### Gene set enrichment analysis

We conducted gene set enrichment analysis (GSEA) using EnrichR [66] with the Mammalian Phenotype Ontology database [57] and the differentially expressed genes appearing in the TAM network. The result was visualized in R using ggplot2 and ComplexHeatmap [17].

### Network-informed target selection for drug repurposing

We calculated features of the network’s topology with Cytoscape’s “Analyzer” app [2]. Two topological features were carried on: the node degree, or number of node interactions, and the betweenness centrality, which accounts for the number of shortest pathways including the node. Further, we queried the TAM network for regulatory motifs using the Cytoscape app “NetMatchStar” [54]. We decided to include four types of feedback and three types of feed-forward loops, a strategy that was used previously to identify network motifs [5] (Supplementary Table 1).

To detect the most important nodes and their interactions, we calculated a weighted score of the identified motifs with the following equation:

$$Scor{e_i} = {w_1} \cdot F{C_i} + {w_2} \cdot B{C_i} + {w_3} \cdot {D_i}$$

The score is based on the method published by Khan et al. [26]. For each motif *i*, the score is calculated with different weight settings for *w*_*1*_, *w*_*2*_, and *w*_*3*_ that define the relative importance of the three motif features: *FC*_*i*_ is motif *i*’s average log_2_FC expression in UM TAM scRNA-Seq data, while *BC*_*i*_ and *D*_*i*_ are motif *i*’s average betweenness centrality and node degree, respectively. The weighting factors sum up to 1 and *w*_*1*_ was fixed at 0.5 to give equal relevance to expression and topological features. We iterated *w*_*2*_ from 0.05 up to 0.45 in 0.05 steps and set *w*_*3*_ to complement to 0.5 (*w*_*3*_ = 0.5 – *w*_*2*_), calculating the motifs’ scores for each iteration. Next, we extracted the Pareto frontier of a motif’s scores with the “psel” method using the R package rPref (version 1.3), as described previously [5, 26].

We considered the nodes represented in the 100 highest-scoring motifs as the core regulatory machinery in TAMs [5]; Khan et al., [26] and extracted them and their interactions to create a core network. This network can be browsed and downloaded from www.vcells.net/TAM-drug-repurposing.

To shortlist the most promising targets for immunomodulation, we removed candidates with expected shortcomings in specificity. This included all genes with reduced expression in TAMs compared to steady-state macrophages, as well as all housekeeping and transcription factor genes and those not involved in inflammation, according to GeneCards (www.genecards.org).

Thus, we ended up with 14 potential targets, of which three (NLRP3, HMOX1, and CASP1) are associated with the inflammasome and eight (GSTP1, NAMPT, HSP90AA1, B2M, PTGS2, ISG15, CSXCL2, LTA4H) were linked to general inflammation. An overview of the targets and their selection process can be found in Supplementary Table 2.

### Pharmacophore modeling and in-silico screening of drug library

We obtained 3D protein structures corresponding to 13 selected targets from the RCSB Protein Data Bank (www.rcsb.org/pdb; Supplementary Table 2) [1, 3, 16, 18, 22, 30, 34, 37, 38, 52, 53, 56, 61]. ZFP36 lacks a PDB entry and was excluded from our study. We applied standard protein preparation protocols in Biovia Discovery Studio 2022 (DS 2022) to set up each structure for pharmacophore generation. We used the ‘Edit and Cluster Features Tool’ of DS 2022 to generate pharmacophore features for each active site, including the features “Hydrogen Bond Donors and Acceptors” and “Hydrophobic”. We relied on the excluded volume constraints to the best-selected pharmacophore model to highlight potentially forbidden sites for the drug molecules during the screening process.

We screened a library of FDA-approved drugs consisting of 1,615 compounds from the Zinc15 database [59]. The screened drugs were ranked based on their FIT values, which indicates the accuracy of drug alignment within the binding site. We selected drug-target interactions with a FIT value score of at least 0.3, and carried forward drugs that interacted with at least ten of the thirteen target proteins.

### Molecular docking

To further refine the prediction of the most promising drugs interacting with the 13 target proteins, we performed molecular docking using CDOCKER restricted to the predicted binding pockets. To this end, we focused on the binding site used for the pharmacophore modeling of the proteins and performed flexible docking using the CDOCKER program in DS 2022. We then analyzed the drug coverage against all proteins, followed by a ranking of all drug-protein target combinations based on CDOCKER-estimated energy.

### Preparation of macrophages

We isolated human peripheral blood mononuclear cells (PBMCs) from freshly drawn peripheral blood of healthy donors (Uniklinikum Erlangen, Department of Transfusion Medicine and Haemostaseology, Germany) by density gradient centrifugation using human Pancoll (1.077 g/ml) (PAN™ Biotech, Aidenbach, Germany) and a subsequent buffy coat purification. To generate macrophages, we isolated monocytes by adherence to polystyrene in CELLSTAR^®^ cell culture flasks (Greiner Bio-One, Kremsmünster, AUT), removed non-adherent cells and cultured remaining monocytes in the presence of Leucomax^®^ GM-CSF (500 U/µl) (Novartis Pharma, Nürnberg, GER). After 6–7 d of culture, macrophages were detached with EDTA (1 mM) (Sigma-Aldrich^®^, München, GER).

### Life imaging (Incucyte)

In addition to cell viability measurements by flow cytometry, we followed cell death by live-cell microscopy using the Incucyte^®^ Cytotox Green Dye (Sartorius, #4633). MDMs were generated as above and were harvested and counted. Cells were seeded at a concentration of 300 000/ml in a 96-well plate (Corning, #353072) and left to adhere for two hours. Afterwards, cells were either left untreated or treated with 25 µg/ml clindamycin overnight. On the next day, cells were challenged with no, 1 µM, or 10 µM nigericin and Cytotox Green was added to every well. The plate was then imaged every 30 min for 24 h in a live-cell imaging system (IncuCyte SX1, Sartorius). The total green area was assessed with the Incucyte 2022 A software (Sartorius).

### Western blot analysis

We seeded macrophages at 2 × 10^6^ cells/ml in polystyrene Falcon^®^ 24-well plates (Corning^®^ LifeSciences, Corning, USA). Cells were incubated for two hours in the presence or absence of LPS (100 ng/ml). Next, clindamycin (25–50 µg/ml) was added directly to the pre-stimulated cells for 30 min. Finally, we challenged the macrophages for 15 min with nigericin (10 µM). In another set up, cells were incubated for two hours in the presence or absence of clindamycin (25 µg/ml). Next, LPS (0.1 µg/ml) was added as a pre-stimulant for two hours. Then, macrophages were challenged for 24 h with either nigericin (10 µM), monosodium urate (MSU) crystals (200 µg/ml, InvivoGen, tlrl-msu), or ATP (5 mM, InvivoGen, tlrl-atp). MSU crystals and ATP were used as alternative stimulants as nigericin is not a physiological pyroptosis-inducing agent. After incubation, the supernatant was removed and immediately frozen at -80 °C. Cells were lysed in 2% (w/v) SDS lysis buffer (5 mM EDTA, 50 mM Tris/HCl, 150 mM NaCl, 2.2% (wt/vol) SDS) supplemented with cOmplete™ protease inhibitor (Roche Diagnostics, Mannheim, Germany). We removed cell debris by centrifugation (21,382x g, 15 min, 4 °C) and the concentration of total protein in cell extracts was determined with Pierce™ BCA Protein Assay Kit (Thermo Fisher Scientific™, 23227). Culture supernatants as well as lysates were suspended in 4× Laemmli sample buffer including 2-mercaptoethanol (Bio-Rad Laboratories, München, Germany) and denatured for 10 min at 95 °C. Lysates were loaded at equal protein amount and supernatants at equal volume onto an SDS-PAGE (12.5%). To evaluate protein size, Precision Plus Protein™ WesternC™ standard (Bio-Rad Laboratories, München, Germany) was loaded in a separate lane. We transferred size-separated proteins onto nitrocellulose membranes (0.2 μm, Cytiva, Marlborough, USA, #10600011) using the semi-dry TransBlot^®^ Turbo™ transfer system (Bio-Rad Laboratories, München, Germany). Membranes were blocked with 5% milk powder in TBS-T (100 mM Tris/HCl, 150 mM NaCl, 0.1% (v/v) Tween^®^-20) for 1 h at room temperature. Afterwards, we incubated the membranes overnight with primary antibody diluted in 5% (w/v) milk powder in TBS-T at 8–10 °C. Membranes were washed with TBS-T and incubated with the appropriate HRP-conjugated secondary antibody diluted in 5% (w/v) milk powder in TBS-T for 1 h at room temperature. Signals were detected by chemiluminescence using the SuperSignal™ West Dura Extended Duration Substrate or SuperSignal ELISA Femto Maximum Sensitivity Substrate (Thermo Fisher Scientific, Waltham, USA) according to the manufacturer’s instructions on an Amersham ImageQuant 800 (Cytiva, Marlborough, USA). Before re-incubation with additional antibodies, membranes were stripped with the Restore Western Blot Stripping Buffer (Thermo Fisher Scientific, Waltham, USA) and blocked again as described above. Primary antibodies used in the study were mouse-anti-GAPDH (1:2000, CellSignaling, #97166) and rabbit-anti-gasdermin D (1:1000, CellSignaling, #97558), secondary antibodies HRP-conjugated horse-anti-mouse IgG (1:2500, CellSignaling #7076) and goat-anti-rabbit IgG (1:2500, CellSignaling, #7074).

### ELISA

We examined cell culture supernatants for human IL-1β and IL-18 with ELISA kits from R&D Systems^®^ (Minneapolis, USA) according to the manufacturer’s instructions.

### LDH release assay

We plated MDMs in 96-well culture at a concentration of 5 × 10^4^ cells/well and pre-activated them with or without LPS (100 ng/ml) for 24 h. Subsequently, we challenged macrophages with nigericin (10 µM) in the presence or absence of clindamycin (10 µg/ml) overnight. LDH released into the supernatant was quantified using CyQUANT^™^ LDH Cytotoxicity Assays (Invitrogen, C20301) according to the manufacturer’s instructions. We derived the pyroptosis prevalence with the following equation: [(experimental release − spontaneous release)/(maximum release − spontaneous release)] × 100, where spontaneous release refers to untreated macrophages and maximum release was obtained by complete lysis of macrophages with a solution of 0.1% Triton X-100.

### FLICA^®^ 660 caspase-1 assay

We detected caspase-1 activity using the FLICA^®^ 660 caspase-1 assay kit from ImmunoChemistry Technologies (Bloomington, USA) according to the manufacturer’s instructions. We seeded macrophages at 1 × 10^6^ cells/ml in polystyrene Falcon^®^ round bottom tubes (Corning^®^ LifeSciences, Corning, USA) for flow cytometry. Cells were LPS-primed (100 ng/ml, 24 h) and challenged overnight with 10 µM nigericin in the presence or absence of clindamycin (10 µg/ml or 25 µg/ml). We washed the cells with PBS and incubated with the FLICA^®^ 660-YVAD-fmk reagent (1:150 v/v, 30 min) at 37 °C and 5% CO2. As assessed by flow cytometry, caspase-1 activation was defined as increase in red fluorescence.

### FLICA^®^ 660 caspase-1 assay of TAMs

After thawing frozen MM patient bone marrow PBMCs at 37 °C, we washed them with PBS and seeded 2.5–5 × 10^6^ cells/ml in polystyrene Falcon^®^ round bottom tubes (Corning^®^ LifeSciences, Corning, USA) for flow cytometry. Cells were either kept untreated or incubated with 25 µg/ml clindamycin (Selleckchem, USA, #S2830) for 24 h. Subsequently, we measured the caspase-1 activity with the FLICA^®^ 660 caspase-1 assay kit from ImmunoChemistry Technologies (Bloomington, USA) according to the manufacturer’s instructions. We washed the cells with PBS (350x g, 4 °C, 4 min) and incubated them with FLICA^®^ 660-YVAD-fmk reagent (1:150 v/v, 60 min) at 37 °C and 5% CO_2_. Afterwards, cells were washed again and counterstained on the surface with CD163-BV421 (BioLegend, USA, #333612) and CD15-BV510 (BioLegend, USA, #323028) for 15 min at 4 °C. Cells were washed again and assessed by flow cytometry. Caspase-1 activation was defined as increase in red fluorescence.

## Results

### Transcriptomics-supported network analysis yields molecular targets linked to immunomodulation of TAMs

We hypothesized that immunomodulation of TAMs in UM can remodel the tumor’s microenvironment and increase its therapy susceptibility. To find molecular targets for immunomodulation, we (a) constructed a comprehensive signal transduction network reflecting the biology of TAMs, (b) projected TAM RNA-seq data into the network nodes and quantified their topological importance, and (c) used scRNA-seq data and topological features to extract a core network including the most connected and differentially expressed genes. From this core, we selected a list of druggable proteins for further investigation.

**Fig. 2:**
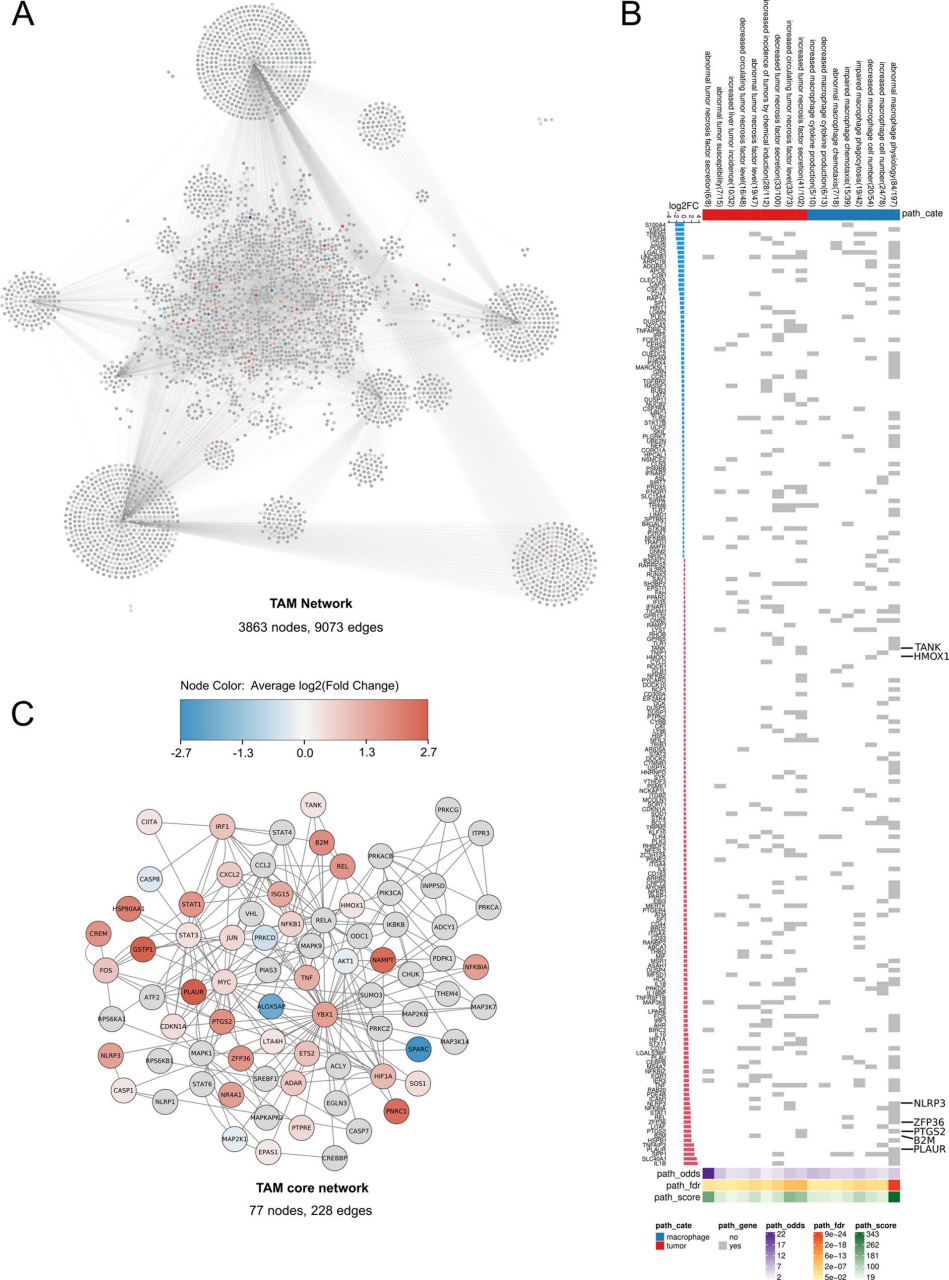
Computational analysis identifies high-importance signal mediators in TAMs as potential targets for drug repurposing. **(A)** TAM-customized pathway network. The network was reconstructed from literature and interaction databases, and tailored towards TAMs with bulk RNA-seq expression data, encompassing 3863 nodes and 9073 edges. (**B)** Heat map visualizing gene-set enrichment analysis results on 927 network genes differentially expressed between TAMs and steady-state macrophages. A gray grid cell in the heat map indicates that a gene is enriched in a pathway. Only tumor- (red) and macrophage-related (blue) pathways are shown. In addition, we annotated each pathway with its odds ratio (odds), false discovery rate (FDR), and an aggregate score (odds x [-log10(FDR)]). The left-side bar plot shows the genes’ corresponding log2FC. We highlighted *TANK*, *HMOX1*, *NLRP3*, *ZFP36*, *PTGS2*, *B2M*, and *PLAUR* because they were later selected for pharmacophore modeling. **(C)** TAM core network extracted through multicriteria ranking of regulatory motifs. The core consists of 77 nodes and 228 edges. Nodes were colored according to their differential expression (log2FC) between TAMs and steady-state macrophages. Nodes in grey showed no significant differential expression

**Fig. 3:**
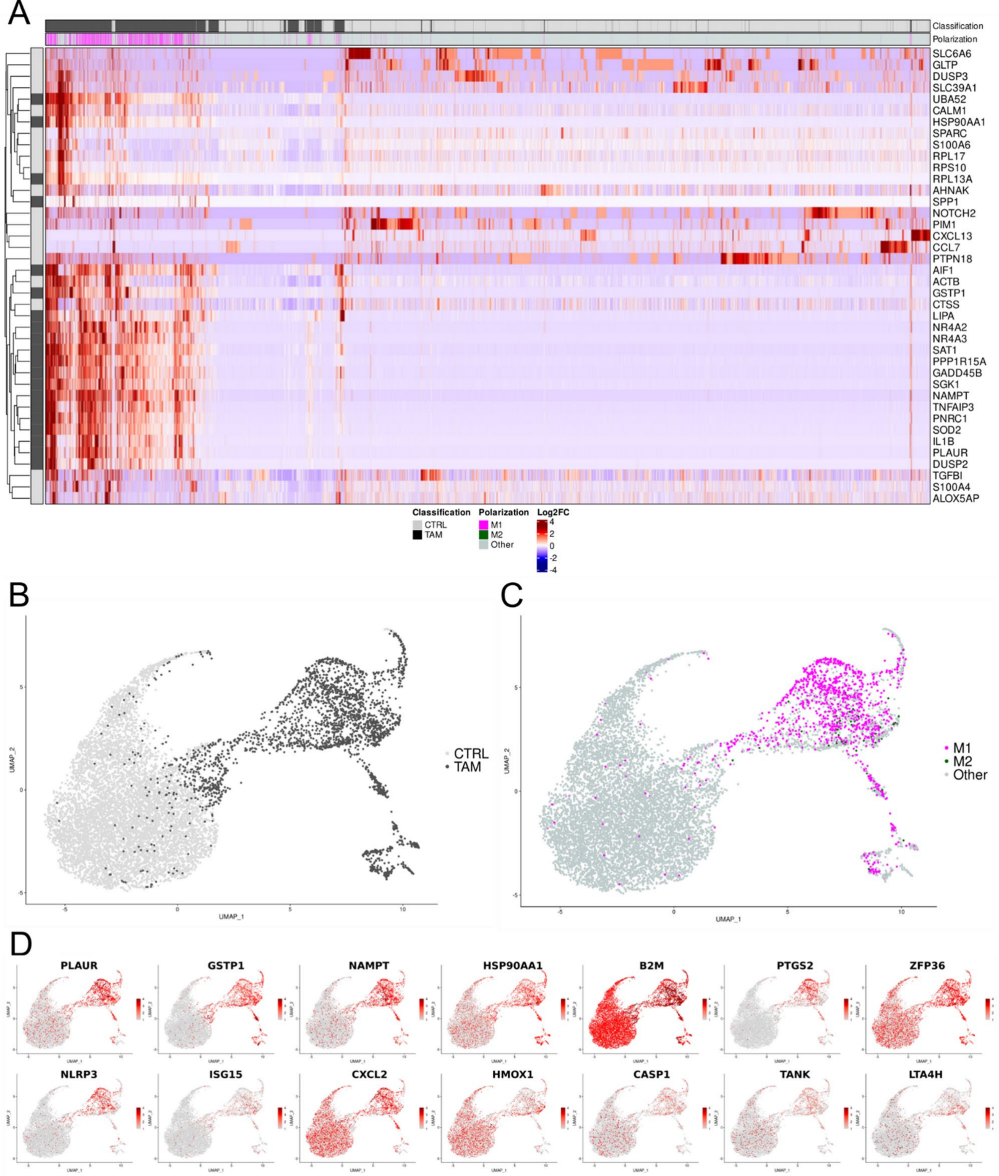
Single-cell expression comparison against steady-state macrophages reveals tumor-associated macrophage (TAM) polarization. **(A)** Heat map of top 40 differentially expressed genes. Column dendrogram omitted. **(B)**,** (C)** UMAP of the combined, batch-corrected data set colored by tissue origin (**B)** or macrophage polarization **(C). (D)** Gene expression of 14 targets selected for pharmacophore modeling. Several targets (e.g., *PTGS2*, *NLRP3*, and *GSTP1*) showed distinct expression in TAMs, while others (e.g., *ZFP36*,* CXCL2*, and *CASP1*) were active to some extent in both TAM and CTRL cells, indicating their role in maintaining homeostasis

- **TAM network reconstruction**. To reconstruct a model of TAM biology, we adapted the pathway machinery of a previously published macrophage signal transduction network [63] to that of TAMs according to literature knowledge, gene expression data, and interaction databases, obtaining a fully-connected graph with 3863 nodes and 9073 edges (Fig. 2A).
- **Integration of TAM-derived scRNA-seq data into the network**. For the ranking procedure in the next section, we annotated the nodes with results from a differential scRNA-seq analysis comparing TAMs and steady-state macrophages. Cells accepted in the analysis amounted to 2656 in the TAM group and 7542 in the steady-state macrophage group (Fig. 3A, B).

The joint data set comprised 35,227 features in total and 2410 differentially expressed features. Of the latter, 927 genes were shared with the TAM network, with 574 genes upregulated in TAMs. To elucidate the polarization of the macrophages, we used M1 polarization markers CD14 and IL-1β and M2 polarization markers CD163 and IL-10 to classify the cells. A look at the regulation pattern of the top 40 differentially expressed genes shows a clear TAM upregulation of inflammatory proteins like IL-1β, NR4A2-3, or TNFAIP3 (Fig. 3A). This result is in line with the almost exclusive M1-like polarization of the macrophages derived from the tumor microenvironment (Fig. 3A, C).

These findings are further corroborated by the generally upregulated pathways observed in the GSEA of the 2410 differentially expressed genes. On the one hand, we found several enriched pathways related to the physiology of macrophages including phagocytosis, chemotaxis, and cytokine production (Fig. 2B). On the other hand, we found several enriched phenotypes associated to the tumor microenvironment, such as tumor necrosis factor secretion. The distribution of all enriched pathways can be found in Supplementary Figure S1.

c. **Core network extraction and target selection**. We assumed that motifs like feedback and feedforward loops play a pivotal role in the (de)regulation of gene networks. Targeting differentially expressed, highly connected and intertangled regulatory motifs, we first extracted all 9035 feedback and feedforward loops with at most 4 nodes from the network (Supplementary Table 1). We then calculated the average per extracted motif of the topological features node degree and node betweenness centrality, as well as the expression feature log_2_ fold change. We combined these metrics into a computational score and used it to extract a core network of Pareto-optimized, top-ranked network motifs with 77 nodes and 228 edges (Fig. 2C). After disregarding transcription factors and known housekeeping genes, we chose the most suitable candidates for pharmacophore modelling based on their degree of overexpression in TAMs, their known role in inflammation, and their link to the inflammasome. This resulted in 14 candidates which were higher expressed in the TAM cell group (Fig. 3D and Supplementary Table 2).

### Pharmacophore modeling and molecular docking highlight Iohexol and clindamycin for repurposing in TAMs

**Fig. 4:**
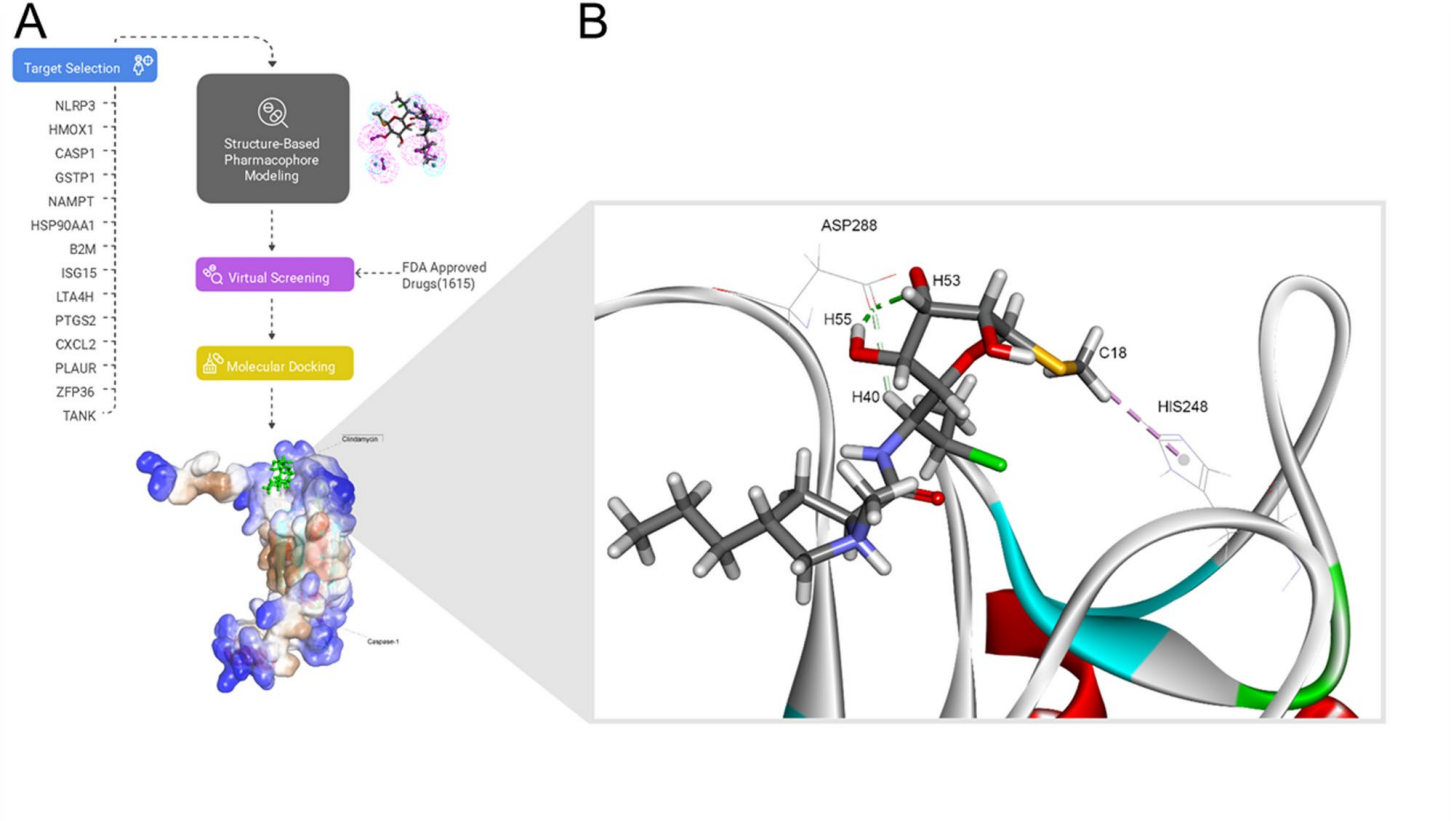
Computational screening of chemical compounds against TAM-expressed protein targets identifies the antibiotic clindamycin as a TAM immunomodulator. **(A)** Pharmacophore modeling and screening of FDA-approved drugs. Except for ZFP36, protein structures of the selected targets were processed to derive pharmacophore models representing potential binding sites. FDA-approved drugs were then subjected to virtual screening, followed by refinement of promising binders using flexible molecular docking. This process led to the identification of the clindamycin-*CASP1* interaction. **(B)** Predicted interactions of clindamycin and *CASP1* residues in the binding pocket

To screen approved drugs against the 14 selected protein target candidates, we obtained their 3D structures from public repositories and prepared pharmacophore models of the relevant binding pockets for each one (Fig. 4A). ZFP36 was discarded since no 3D structure was available.

Docking experiments were performed to identify drugs with promising target interactions. During these experiments, five targets (LTA4H_8AVA, HSP90AA1_5J2X, NLRP3_3QF2, PLAUR_1YWH, and TANK_1KZZ) failed to bind any drugs and were excluded. The remaining eight target exhibited interactions with ZINC000003830943 and ZINC000003830944. These two compounds are constituents of the drug iohexol, a water-soluble contrast agent. Meanwhile, ZINC000003978028 (antibiotic clindamycin) interacted with seven targets, as detailed in Supplementary Table 3. To further evaluate the compounds and their interaction with the targets, we estimated binding energies through molecular docking (Supplementary Table 4). Notably, both clindamycin and iohexol demonstrated interactions with two otherwise undockable target proteins (iohexol with *NAMPT*, clindamycin with *CASP1*), and both drugs bound to all other six targets when the best-docked cases were selected, as summarized in Supplementary Table 5. However, iohexol is not taken up by cells and, therefore, unsuitable for direct modulation of intracellular processes [48]. Thus, clindamycin and its interaction with cascape-1 emerged as the best choice for experimental validation. Figure 4B and Supplementary Table 6 contain further information concerning the binding and interaction of clindamycin to caspase-1.

### *In vitro* immune challenge confirms that clindamycin reduces pyroptotic cell death in human monocyte-derived macrophages (MDMs)

**Fig. 5:**
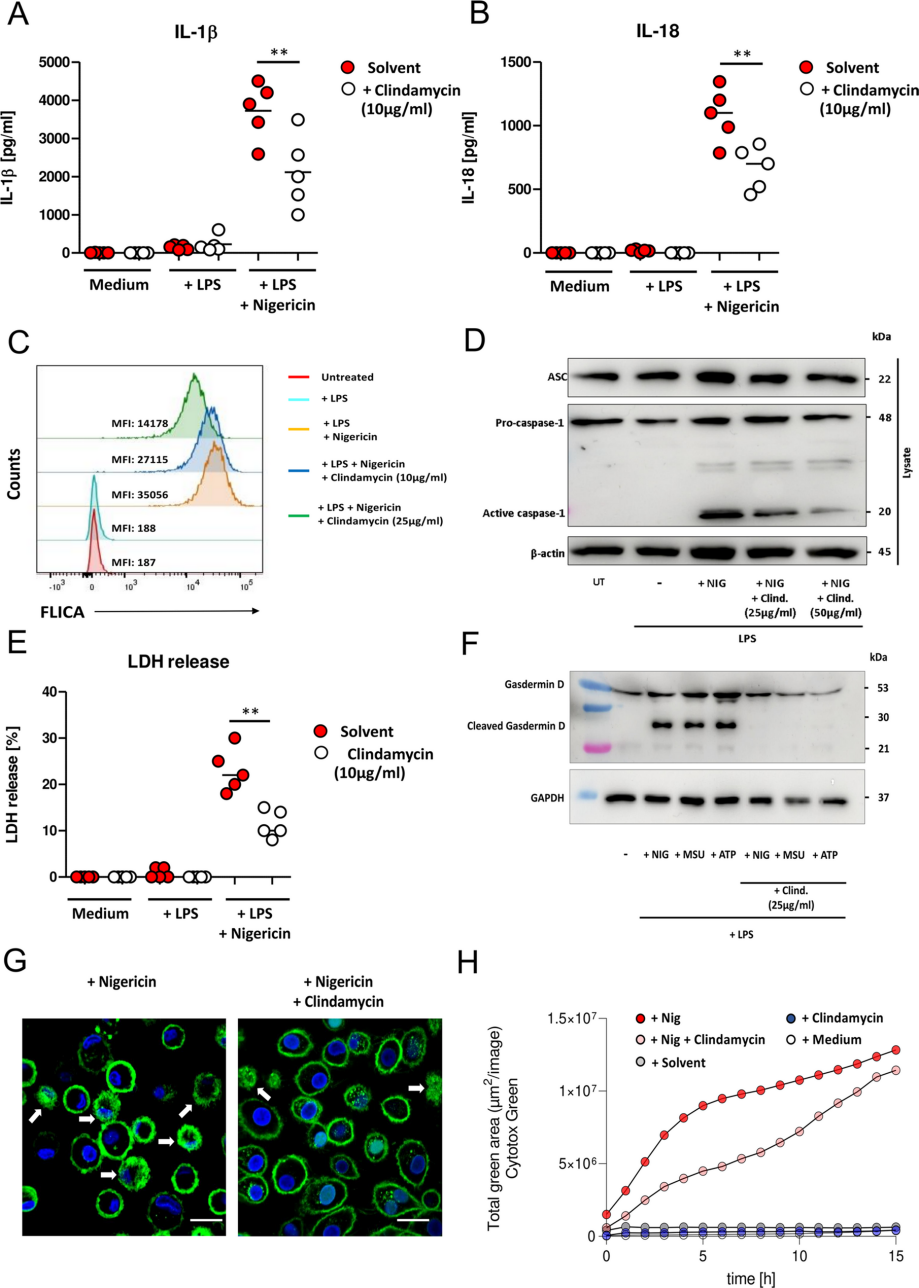
Clindamycin suppresses the pyroptotic phenotype in monocyte-derived macrophages (MDMs). MDMs were incubated with (white circle) or without (red circle) clindamycin (2 h, 10 µg/ml) before being exposed to LPS (100 ng/ml) alone or in combination with nigericin (10 µM) for 3 h. Supernatants were assayed for IL-1β (**A**) and IL-18 (**B**) by ELISA. **(C)** MDMs were treated as indicated and caspase-1 activation was estimated as Mean Fluorescence Intensity (MFI) using FLICA by flow cytometry. (**D**) MDMs were treated as indicated and caspase-1 activation was measured by Western blotting. The band at a height of 20 kDa is the cleaved active caspase-1, while ASC and GAPDH served as controls in the same lysates. (**E**) MDM were treated with LPS (100 ng/ml) and nigericin (10 µM) in the presence or absence of clindamycin, and supernatants were assayed for LDH release after 24 h. (**F**) MDM were treated as indicated and cleavage of gasdermin D was measured by Western blotting. The band at a height of 30 kDa is the cleaved, active form of gasdermin D, and ß-actin serves as a loading control in the same lysates. (**G**) MDM were incubated with or without clindamycin (25 µg/ml, 18 h) and then challenged with nigericin (10 µM, 3 h). To investigate the membrane structure, macrophages were stained with Wheat Germ Agglutinin (WGA, green). DAPI (blue) was used as a counterstain for nuclei. The arrows indicate cell membrane damage. Scale bar: 10 µM. (**H**) MDM were incubated with or without clindamycin (25 µg/ml, 18 h) and then challenged with nigericin (10 µM) for 15 h. Cell membrane integrity loss was measured as uptake of Cytotox Green dye under a live imaging microscope (Incucyte). The graph shows the increase in green fluorescent area (µm^2^/image) over the challenge period

The inflammasome and in particular its constituent NLRP3 have been implicated in -tumor progression [19, 21]. Taking advantage of the fact that NLRP3 is one of caspase-1’s upstream activators, we employed a nigericin challenge assay to assess the effect of clindamycin on NLRP3-inflammasome activation in human macrophages. Nigericin is a microbial toxin that triggers the NLRP3 inflammasome-mediated maturation and release of IL-1β and IL-18 [25]. After challenge of LPS-preactivated MDMs with nigericin for 24 h, we observed an increase in IL-1β and IL-18 in the supernatant (Fig. 5A-B). The addition of clindamycin showed a significant reduction in nigericin-induced secretion of IL-1β and IL-18 (Fig. 5A-B), suggesting that clindamycin suppresses canonical inflammasome activity.

To test whether clindamycin specifically interacts with the active site of caspase-1, we measured caspase-1 activation in nigericin-treated macrophages using FLICA^®^ reagent, a cell-permeant fluorescent-labeled inhibitor binding specifically and covalently to active caspase-1 [21]. Flow cytometry showed an increase in FLICA-derived fluorescence signal in macrophages upon nigericin challenge in comparison to LPS-preactivated macrophages (Fig. 5C, signal-level quantification in Figure S3). Clindamycin co-application led to a dose-dependent reduction in fluorescence (up to a 2.5-fold reduction, Fig. 5C), suggesting that clindamycin competes with FLICA for binding. To determine whether clindamycin binding interferes with caspase-1 activation, we monitored cleavage of caspase-1 by Western blot analysis. Nigericin challenge triggered cleavage of pro-caspase-1 into its active form p20, while pretreatment with clindamycin decreased the nigericin-induced rise in caspase-1 p20 in a dose-dependent manner (Fig. 5D, signal-level quantification in Figure S3). In the same cell lysates, we did not observe any differences in the amount of the adapter molecule ASC, which recruits pro-caspase-1 for activation. Together, this suggests that clindamycin binds to caspase-1, blocking its active site.

Since challenge with nigericin ultimately results in macrophage pyroptosis, of which caspase-1 is a key mediator, we speculated that clindamycin may suppress pyroptotic cell death. To test this, we challenged macrophages with nigericin in the presence or absence of clindamycin, and measured LDH release into the supernatant after 24 h by ELISA (Fig. 5E). Nigericin challenge resulted in elevated LDH levels, which were attenuated in the presence of clindamycin. Next, we investigated the cleavage of gasdermin D (GSDMD) because it plays a key role in pyroptosis and the proteolytic activation of GSDMD is primarily mediated by caspase-1. While nigericin challenge lead to increased amounts of cleaved, active GSDMD (30 kDa), clindamycin co-treatment resulted in a significant reduction of the cleavage product (Fig. 5F, signal-level quantification in Figure S4). In addition, clindamycin also inhibited GSDMD activation by other pathological stress inducers, such as extracellular ATP and monosodium urate (MSU) crystals (Fig. 5F, signal-level quantification in Figure S6).

Since cleaved GSDMD forms membrane pores that promote cytolysis, we next analyzed the membrane structure of challenged macrophages using confocal microscopy. While nigericin challenge induced a ruffled membrane appearance, pre-incubation with clindamycin effectively blocked this (Fig. 5G). Furthermore, live imaging microscopy showed that macrophages challenged with nigericin undergo membrane integrity loss consistent with pyroptotic cell death, but pre-incubation with clindamycin slowed this development (Fig. 5H). These findings are in line with a caspase-1-specific inhibitory effect of clindamycin able to reduce pyroptosis in macrophages *in vitro*.

**Fig. 6:**
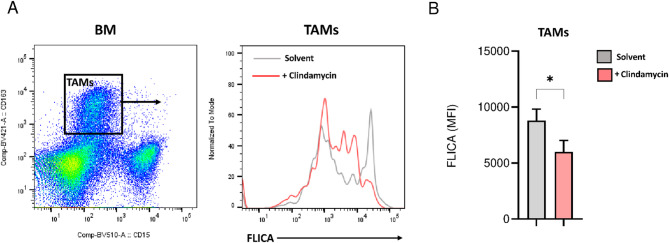
Inflammasome hyperactivity in multiple myeloma TAMs is attenuated by clindamycin. Bone marrow (BM) from MM patients with initial diagnosis was incubated in the presence (25 µg/ml, red) or absence (grey) of clindamycin for 18 h. Subsequently, the activity of caspase-1 in TAMs (CD163+/CD15-) (**A**, left) was analyzed using FLICA by flow cytometry (**A**, right). (**B**) Quantification of FLICA signal from (A) in material from six patients. Caspase-1 activity was estimated as Mean Fluorescence Intensity (MFI). Two-tailed paired Student’s t-test was performed. Error bars show SEM

We have previously shown that TAMs from multiple myeloma (MM) patients exhibit an overactive inflammasome-caspase-1-axis [21], which drives the progression of the disease. To test clindamycin’s effect on this axis in a disease context, we incubated bone marrow (BM) biopsies from MM patients (*n* = 6) with clindamycin and quantified activated caspase-1 using FLICA by flow cytometry. While untreated TAMs (gated as CD163+/CD15-) displayed high levels of activated caspase-1, clindamycin treatment led to a significant reduction (Fig. 6).

Taken together, these results indicate that the predicted interaction between clindamycin and caspase-1 has the potential to counteract TAM pyroptosis-driven tumor promotion.

## Discussion

TAMs are prominent infiltrating immune agents in UM liver metastases, and their abundance positively correlates with an unfavorable prognosis [29, 62]. Given the limited success of current therapies in improving outcomes for UM patients with liver metastases [64], we hypothesized that drug-based immunomodulation of UM-associated macrophages has the potential to remodel the tumor microenvironment, and, in the long run, enhance the effectiveness of therapeutic interventions.

In our workflow, we first created a regulatory network involving protein and gene interactions linked to TAM biology. To adapt the network to genes specific for UM, we compared scRNA-Seq data of UM TAMs and steady-state macrophages and derived a small, highly-interconnected core network. This procedure can be further improved by using a more comprehensive collection of UM-associated macrophages and other tissues of origin for our control macrophages. Surprisingly, the polarization status of most macrophages from the UM environment was classified as M1 in the analyzed single-cell data. This contradicts previous studies reporting mainly M2 polarization in UM TAMs [23]. However, TAM research has gradually moved away from the simplified model of M1/M2 polarization, and UM TAMs are no exception [36].

From the genes belonging to the core network, we followed a stringent procedure to select the most promising protein targets, characterized by overexpression in TAMs compared to steady-state macrophages, cellular localization, and whether their predicted impact on immune-related pathways enables immune modulation. We then performed a pharmacophore screening of FDA-approved drugs to identify novel putative interactions with the selected genes. After a refinement step with flexible molecular docking and further additional ranking steps, we decided to focus on the clindamycin-caspase-1-interaction for experimental validation.

Clindamycin is a bacteriostatic or bacteriocidal antibiotic that interferes with bacterial protein synthesis. Recently, antibiotics have been proposed as repurposable drugs for cancer, and several clinical trials are investigating their efficacy as anticancer therapy [50]. Caspase-1 participates in the execution phase of apoptosis and has been found to potentiate the pro-tumor effect of TAMs [8, 12, 33, 35, 39, 44, 49]. It plays a pivotal role as a key effector in the pro-inflammatory response triggered by inflammasome activation [58]. In the inflammasome, activated NLRP3 recruits the adaptor molecule ASC, which binds to pro-caspase-1 and triggers its autocatalytic activation. Active caspase-1 then cleaves the pro-cytokines IL-1β and IL-18, activating and preparing them for release [32]. Further, caspase-1 activation can trigger pyroptosis, a programmed immune cell death characterized by plasma-membrane rupture and release of pro-inflammatory intracellular content [43]. Pyroptosis in the tumor microenvironment supports a chronic inflammatory milieu that enhances cancer cell transformation and promotes immune escape [24]. Moreover, recent findings report the blockade of IL-1β-signaling to induce TAM reprogramming and decrease inflammation [6]. With this in mind, we sought experimental confirmation of clindamycin’s inhibition of the NLRP3/caspase-1 axis and pyroptosis [10]. On a side note, both caspase-1 and NLRP3 were shortlisted as preferential targets in our network analysis (Sup. Table 2).

We found that clindamycin indeed suppressed inflammasome activation-induced secretion of IL-1β and IL-18 in LPS-pre-activated macrophages, which were challenged with nigericin and other NLRP3-activating substances. Our data further indicates that this effect occurs downstream of ASC in the NLRP3-ASC-caspase-1 signaling pathway, suggesting that clindamycin acts directly on caspase-1. Finally, we used confocal microscopy, LDH release assays, and Gasdermin D cleavage assays to confirm that clindamycin-mediated inhibition of caspase-1 reduced pyroptosis in macrophages. We resorted to using macrophages derived from peripheral blood monocytes isolated from healthy donors as obtaining viable TAMs from UM and its metastases is challenging due to the rarity of this tumor entity. To redeem this shortcoming, we took advantage of ex vivo TAMs in bone marrow biopsies from multiple myeloma patients after initial diagnosis. Also in this second experimental setup, the clindamycin-mediated inhibition of caspase-1 activity was observed, underscoring the robustness of the earlier findings. We hope that further preclinical in vivo experiments with animal models will corroborate the anti-inflammatory effect of clindamycin on TAMs.

## Conclusions

In conclusion, we propose a network-supported pipeline for drug repurposing that allows for filtering and prioritization of drug-target interactions. With this method, we predicted and validated a new drug-target interaction that efficaciously blocks caspase-1-mediated inflammasome activity *in vitro*. This new clindamycin interaction holds potential for the immunomodulation of TAMs in metastatic uveal melanoma and other cancers.

## Electronic supplementary material

Below is the link to the electronic supplementary material.

Supplementary Material 1

## Data Availability

No datasets were generated or analysed during the current study.

## Abbreviations

UM: Uveal melanoma
TAM: Tumor-associated macrophages
LDH: Lactate dehydrogenase
scRNA-Seq: single-cell RNA sequencing
FC: Fold-change
TPM: Transcripts per million
BC: Betweenness centrality
D: Degree
NCBI: National Center for Biotechnology Information
GEO: Gene Expression Omnibus
HRP: Horseradish peroxidase
GSDMD: Gasdermin D
BM: Bone marrow
MM: Multiple myeloma
MSU: Monosodium urate
LPS: Lipopolysaccharide
