# Supplementary material for: Repurposed clindamycin suppresses pyroptosis in tumor-associated macrophages through Inhibition of caspase-1
Source: J Exp Clin Cancer Res. 2025 Aug 4;44:225. doi: 10.1186/s13046-025-03478-5 (PMC12320367; doi:10.1186/s13046-025-03478-5)
Supplement: Supplementary file 1 — Supplementary Material 1 [file 13046_2025_3478_MOESM1_ESM.docx]

Repurposed clindamycin suppresses pyroptosis in tumor-associated macrophages through inhibition of caspase-1

**Supplementary Material**


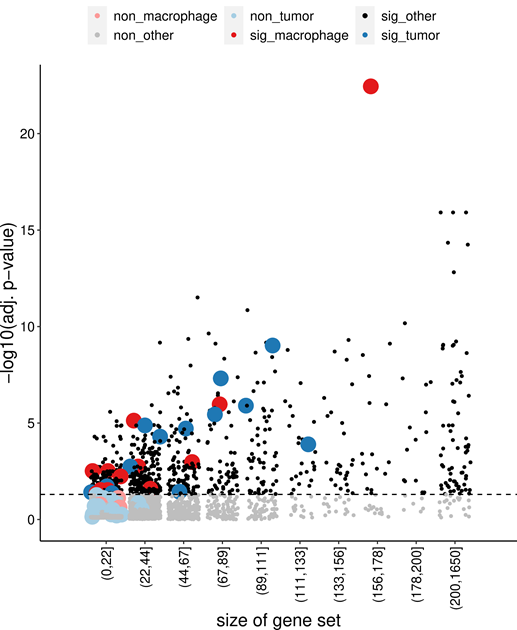


**Figure S1. Distribution of the sizes of gene sets with significantly enriched phenotypes in the GSEA.** The gene sets are classified into the corresponding groups (x-axis) based on their sizes, and the tick label is an interval representing the minimum and maximum gene sizes in a group. The y-axis is the negative value of the log10 transformation of the adjusted p-value, and the gene sets with adjusted p-values greater than 0.05 (below the dashed line) are shaded. Macrophage-related and tumor related gene sets are highlighted in red and blue, respectively.


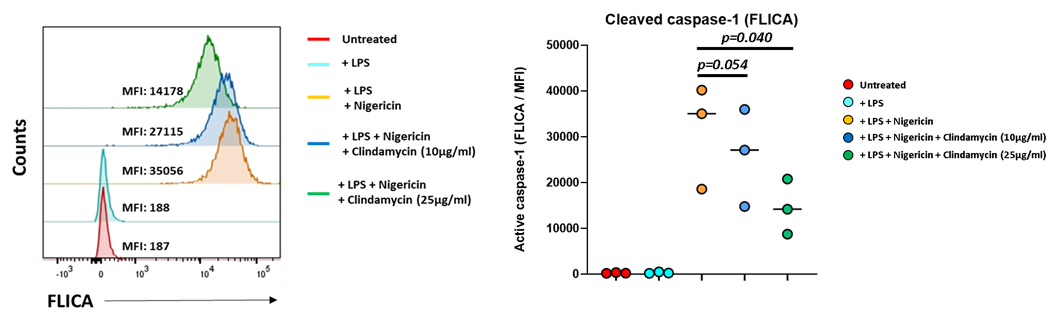


**Figure S2. Triplicate experiments FLICA.** Exact activate Caspase-1 levels as detected by FLICA by flow cytometry. Refers to Figure 5 C (left).

**Figure S3. Western Blot with signal intensity levels.** Refers to Figure 5 D (left).


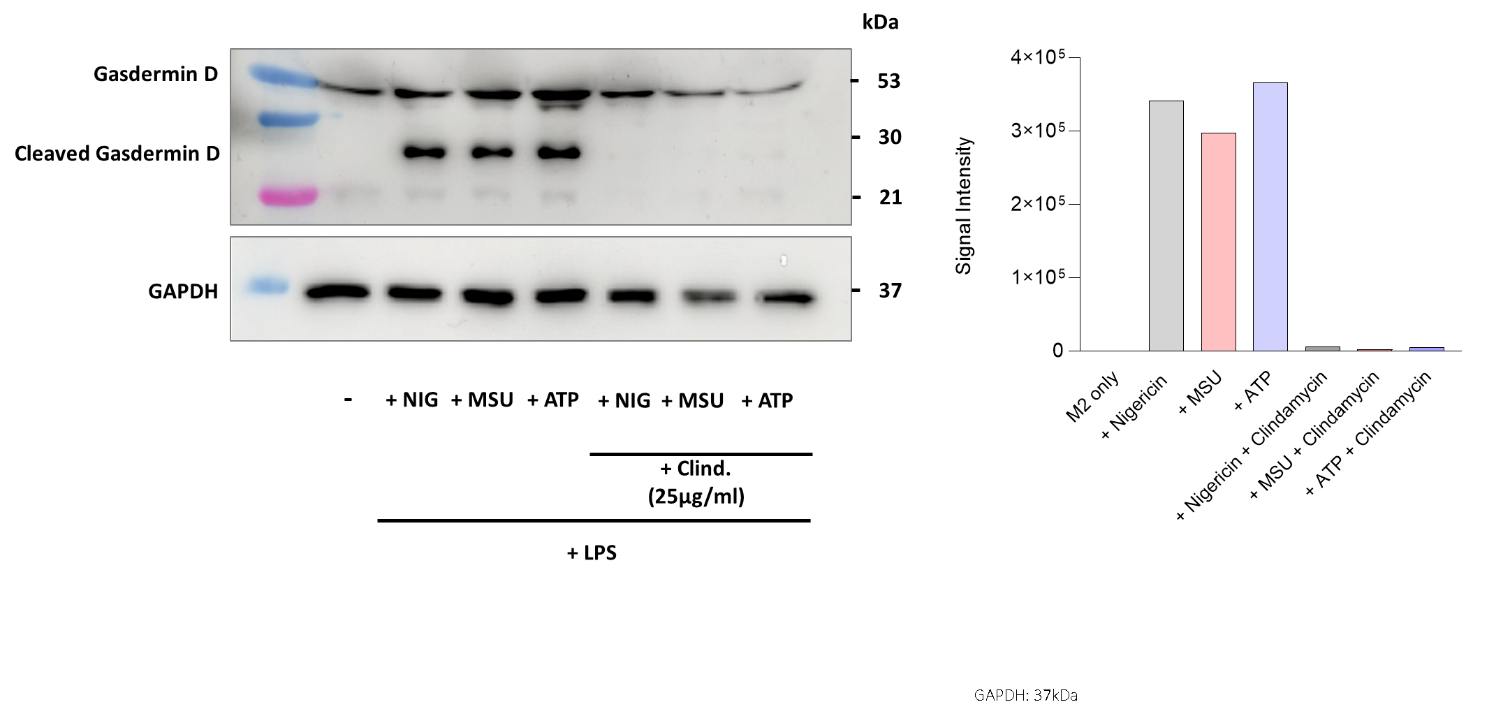


**Figure S4. Western Blot with signal intensity levels.** Refers to Figure 5F (left).

**Supplementary Table 1. Overview of TAM network motif detection.**

| **Motif** | **Occurrences** |
| --- | --- |
| 2-nodes-2-edges Feedback Loop | 30 |
| 3-nodes-3-edges Feedback Loop | 25 |
| 3-nodes-4-edges Feedback Loop | 14 |
| 4-nodes-4-edges Feedback Loop | 65 |
| 3-nodes-3-edges Forward Loop | 2990 |
| 3-nodes-4-edges Forward Loop | 270 |
| 4-nodes-4-edges Forward Loop | 5641 |

**Supplementary Table 2. Target selection based on core network and expression data.**

TF ?

Housekeeping?

Log2 FC > 0?

**50 top scored network nodes**


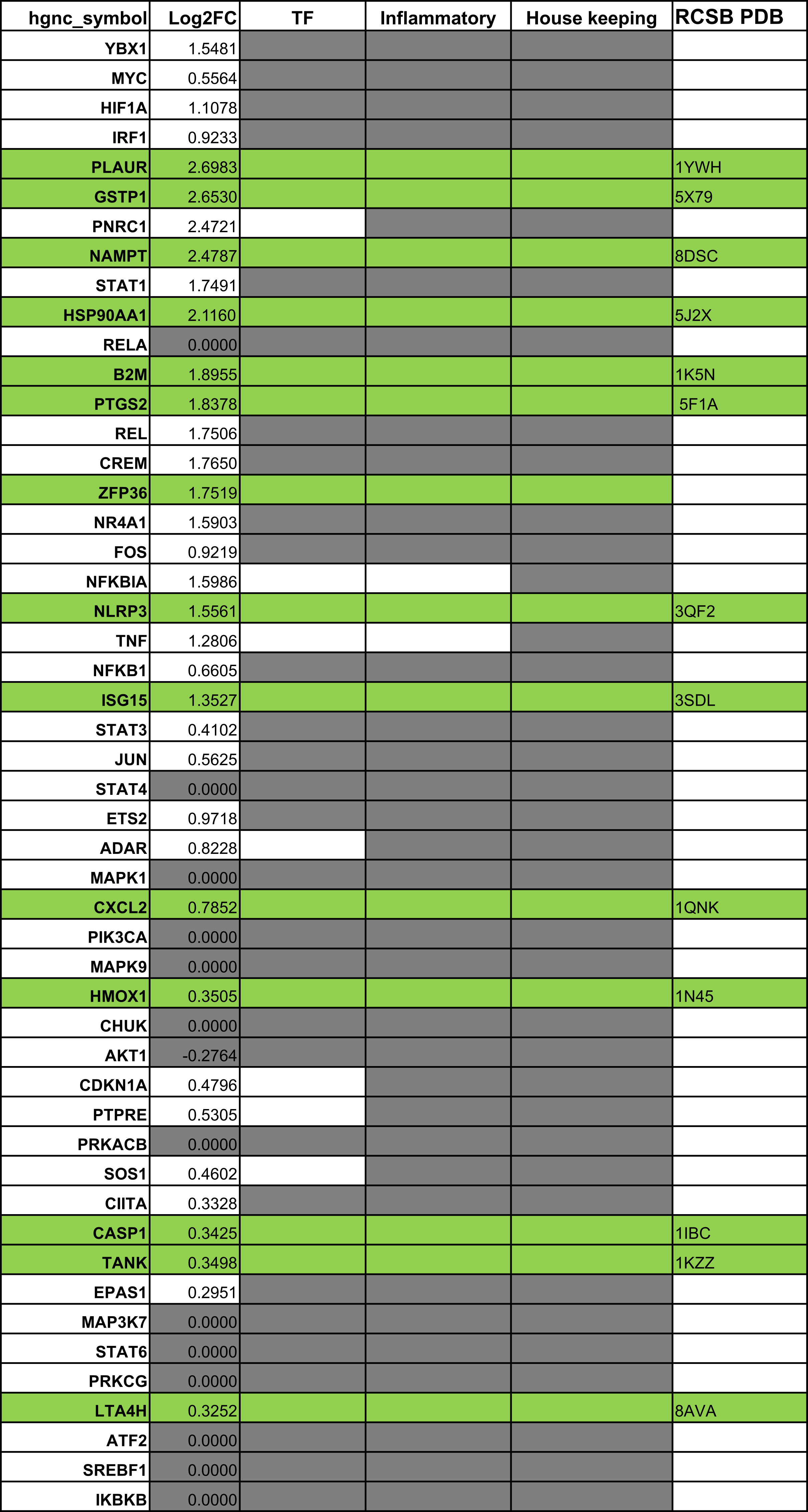


Yes (37 nodes)

No (22 nodes)

Inflammation?

Yes (17 nodes)

No (14 nodes)

**Pharmacophore modelling**

**Supplementary Table 3. Summary of the drugs identified through CDOCKER filtering.** We highlight ZINC000003830943 and ZINC000003830944, which exhibited interactions with eight target proteins. Additionally, ZINC000003978028 (Clindamycin) demonstrated docking interactions with seven target proteins.

| **ZINC ID** | **No. of Target** | **PDB ID and Name of the protein** |
| --- | --- | --- |
| ZINC000003830943 | 8 | B2M_1K5N, CASP1_1IBC, CXCL2_1QNK, GSTP1_5X79, HMOX1_1N45, ISG15_3SLD, NAMPT_8DSC, PTGS2_5F1A |
| ZINC000003830944 | 8 | B2M_1K5N, CASP1_1IBC, CXCL2_1QNK, GSTP1_5X79, HMOX1_1N45, ISG15_3SLD, NAMPT_8DSC, PTGS2_5F1A |
| ZINC000003978028 | 7 | B2M_1K5N, CASP1_1IBC, CXCL2_1QNK, GSTP1_5X79, HMOX1_1N45, ISG15_3SLD, PTGS2_5F1A |

**Supplementary Table 4. Comparison of docking interactions of two drugs—ZINC000003830943/ ZINC000003830944 (Iohexol) and ZINC000003978028 (Clindamycin)—**across various target proteins. Positive CDOCKER energy values were excluded from the analysis, as they indicate weak or unfavorable interactions.

| **Protein Symbol_PDB ID** | **ZINC ID** | **(-) CDOCKER Energy(kcal/mol)** |
| --- | --- | --- |
| B2M_1K5N | Iohexol | 27.779 |
| B2M_1K5N | Clindamycin | 5.139 |
| CASP1_1IBC | Clindamycin | 21.476 |
| CXCL2_1QNK | Iohexol | 21.447 |
| CXCL2_1QNK | Clindamycin | 1.144 |
| GSTP1_5X79 | Iohexol | 14.171 |
| HMOX1_1N45 | Iohexol | 28.990 |
| HMOX1_1N45 | Clindamycin | 0.896 |
| ISG15_3SLD | Iohexol | 0.7278 |
| ISG15_3SLD | Clindamycin | 24.202 |
| NAMPT_8DSC | Iohexol | 24.864 |
| PTGS2_5F1A | Clindamycin | 1.6773 |

**Supplementary Table 5. This table incorporates all relevant parameters, including binding energies, target coverage, and unique target interactions.** The results indicate that ZINC000003978028 (Clindamycin) met all criteria and emerged as the most promising drug candidate.

| **ZINC ID** | **Top drugs based on CDOCKER energy** | **Drugs bind to targets** | **Drugs bind to unique targets** |
| --- | --- | --- | --- |
| Clindamycin | 3 | 6 | 2 |
| Iohexol | 5 | 6 | 2 |

**Supplementary Table 6. Bond information of Clindamycin and with CASP1.**

| **Name** | **Distance** | **Category** | **Types** |
| --- | --- | --- | --- |
| Clindamycin:H53 - CASP1:ASP288:OD1 | 2.21746 | Hydrogen Bond | Conventional Hydrogen Bond |
| Clindamycin:H55 - CASP1:ASP288:OD1 | 2.06199 | Hydrogen Bond | Conventional Hydrogen Bond |
| Clindamycin:H40 - CASP1:ASP288:OD1 | 2.6524 | Hydrogen Bond | Carbon Hydrogen Bond |
| CASP1:HIS248 - Clindamycin:C18 | 5.3007 | Hydrophobic | Pi-Alkyl |
